# Experiences of students with disabilities in technical vocational education and training colleges

**DOI:** 10.4102/ajod.v13i0.1477

**Published:** 2024-11-27

**Authors:** Precious Muzite, Velisiwe Gasa

**Affiliations:** 1 Department of Educational Foundations, College of Education, University of South Africa, Pretoria, South Africa

**Keywords:** students with disabilities, physical disabilities, learning disabilities, technical vocational education and training (TVET), decolonial theory, evidence-based resources (EBRs), evidence-based action

## Abstract

**Background:**

Technical Vocational Education and Training (TVET) in South Africa is often viewed as the ‘Cinderella’ of higher education, with many matriculating students choosing mainstream universities instead. This preference stems from stigma and misconceptions that label TVET students – often from poorer working-class backgrounds – as less intelligent than their university peers. The lived experiences of students with physical and learning disabilities in these institutions are particularly underexplored.

**Objectives:**

This study aimed to illuminate the experiences of students with disabilities at five TVET colleges in Gauteng, South Africa.

**Method:**

Using a phenomenological approach, the study conducted story exercises and individual interviews with a convenience sample of 40 students with disabilities.

**Results:**

The findings reveal that TVET education predominantly serves black students from marginalised backgrounds, with students with disabilities facing significant barriers in accessing both the curriculum and the physical environments of the colleges. Despite numerous challenges, a resilient narrative emerged among these students, rooted in African traditional values.

**Conclusion:**

This article contributes to knowledge on disability inclusion in higher education by showcasing the challenges and resilience of students with disabilities in South Africa’s TVET system.

**Contribution:**

The study employed innovative methodologies, such as picture stories, to co-create knowledge with students living with disabilities.

## Introduction

The main characters in this article are students with disabilities who attend five technical vocational education and training (TVET) colleges in the Gauteng province of South Africa. These students want to be a part of the transformation process, which has been linked to discussions about how higher education institutions from the Global South should look in an advanced new world order (De Sousa Santos 2018; Mbembe 2016; Ndlovu Gatsheni 2013). The article uses a decolonial lens in disability research in education to contextualise the experiences of the students with disabilities enrolled in TVET colleges in South Africa. Decoloniality is a liberating process that has gained popularity in critical race, ethnicity and education politics (Grosfoguel 2004; Mbembe 2016). Scholars such as Meekosha (2011) have raised concerns about how Disability Studies in the education context were previously excluded from the decolonisation process, with most writings on disability coming from ‘western’ countries. Most importantly, the stories of students with disabilities in South African TVET colleges have been left out of the transformation process and the way higher education institutions in the Global South are represented. Most of the existing literature has failed to explore disability inclusion by connecting evidence-informed research with evidence-based action. This therefore sets out a research niche that this study aims to explore. In addition to critically unpacking the lived experiences of the students with disabilities at TVET colleges using an indigenous lens, this study also set out to provide recommendations towards promoting disability inclusion through evidence-informed action.

This article is based on our ongoing postdoctoral research, which looks at the experiences of students with physical and learning disabilities who were enrolled in five TVET institutions within the context of South Africa. Technical vocational education training falls under the Department of Higher Education and Training (DHET) and focusses on vocational and occupational education and training to prepare students to become functional workers in a skilled trade. Universities require students to have a bachelor’s degree at the matriculation level, while TVET colleges accept those who have passed Grades 9, 10, 11 or 12. Some colleges offer up to 300 different courses. These include the National Certificate (Vocational), NATED/Report 191 and NQF Full Time and Learnerships. According to the DHET website (2017), there are 50 registered and accredited public TVET colleges in South Africa, which operate on more than 264 campuses spread across the rural and urban areas of the country. The ‘Context’ section is going to look at the context around inclusive practises in TVET settings and evidence-based resources (EBRs).

### Context

The curriculum of TVET colleges is considerably different from that of mainstream universities in South Africa in the sense that TVET colleges are mostly modelled within a Model Two education system, as described by Dworzanowski and Chagonda (2006) in their study of postgraduate studies at the University of Johannesburg. Model Two systems have been hailed as exceptional as they seek to marry theoretical frameworks with the notion of integrated learning and training. This model is ‘intrinsically trans-disciplinary, trans-institutional and heterogeneous’ (Dworzanowski & Chagonda 2006:2). Integrated learning in TVET colleges commonly fuses theoretical frameworks with the practical part of learning that originally came with an apprenticeship. Apprenticeship is largely absent in Model One frameworks, which are a characteristic of many mainstream universities. Thus, part of the reason TVET colleges have been appealing is their capacity to provide students with the necessary integrated learning in fields such as engineering, boiler making, vocational training and artisanship, manufacturing and technology, services, building construction and security. These fields have been identified by the South African government as critical sectors in the country’s economy.

There has been an increase in the demand for EBRs in research in the Social Sciences (Animasahun et al. 2021). This is particularly true in emancipatory research, which is research aimed at improving the health and livelihoods of marginalised identities. Evidence-based resources are defined as ‘published (multidisciplinary) reviews of intervention evaluations and studies [aimed at] improving health that have evidence of effectiveness, feasibility, reach, sustainability, and transferability’ (US Department of Health and Human Services 2010). Common types that have been used are the systematic-based resources used for Interventions Engaging Community Health Workers in Breast Cancer (Hong et al. 2021) and the non-systematic reviews used scientific evidence briefs, such as those from the National Academies of Science, Medicine and Engineering in the United States of America. In Africa, EBRs have greatly been used in medical fraternities, for example, a systemic evidence-based surgery for trainees of the College of Surgeons of East, Central and Southern Africa (Animasahun et al. 2021). Few studies have carried out systemic literature reviews of both international and local published materials, commissioned research, government policy and related official documents and anecdotal evidence-based disability research to influence positive changes in their lives (Mmatli 2009).

### Literature review

Current literature on disability inclusion in post-secondary training has focussed on five main pillars, that is Universal Design for Learning (UDL) (Ferguson et al. 2019), Inclusive Education and Postsecondary Outcomes (Hughson & Uditsky 2018), Barriers and Facilitators (Taff & Clifton 2022), Technology and Assistive Tools (Lyner-Cleophas 2019) and Community and Social Inclusion (Gidley et al. 2010).

Universal Design for Learning emphasises creating educational environments that accommodate all learners, including those with disabilities (Ferguson et al. 2019). The same study also found that UDL involves flexible teaching methods, materials and assessments to support diverse learning needs.

Lee and Taylor’s (2022) research indicates that inclusive education positively impacts postsecondary outcomes for students with intellectual and developmental disabilities (IDD). Studies show that inclusive education correlates with better employment and educational outcomes (Ndlovu & Walton 2016)

A systematic review identified various barriers and facilitators to disability disclosure and accommodations in post-secondary education. Key barriers include stigma and lack of awareness, while facilitators include supportive policies and accessible resources (Lindsay et al. 2018).

Numerous books and articles have been written about learning and inclusion scholarships in Southern African higher education institutions (Chataika 2009; Bell 2011; Matshedisho 2018). However, most of these studies have referred to traditional universities when they refer to ‘higher education institutions’. An analysis of research on Southern African inclusive pedagogies by Powell and McGrath (2013) in higher education institutions reveals conflicts and some unresolved problems. While most Doctoral and Master’s researchers scramble for research on traditional universities, there appears to be a tacit understanding among academic researchers that research on TVET colleges is not sufficiently ‘academic’ to merit the effort (Powell & McGrath 2013). There is therefore a demand for research that prioritises TVET colleges and most importantly, the inclusion of students with disabilities in these institutions.

The ‘Theoretical framework’ section will unpack why this study’s indigenous epistemology was imperative.

### Theoretical framework

This study is thought to be particularly unique in that it applies the Decolonial Theory to a disability study. To fully appreciate the significance of the voice of people with disabilities, Decolonial Theory may also offer a crucial extension of Critical Disability Theory. It is acknowledged that people without disabilities have been speaking for and misrepresenting people with disabilities and that people with disabilities should have a voice because they have lived experience of disabilities (Devlin & Pothier 2006; Hosking 2008). Under the framework established by decolonial theory, anecdotal evidence and personal experience are valued and serve as crucial tools for appreciating the differing perspectives of diverse, plural and unique individuals (Ndlovu-Gatsheni 2013). Such a theory also emphasises the need to give everyone a voice and the usefulness of all knowledge (Ndlovu-Gatsheni 2013). Because Decolonial Theory makes it possible to identify the underlying causes of certain problems faced by students with disabilities and offers solutions, the current study may be able to provide both an empirical and theoretical contribution.

## Research methods and design

A phenomenological research methodology was used in this study, drawing upon five selected case studies of TVET colleges and campuses around Johannesburg. Individual semi-structured interviews were carried out with 40 youths with a broad range of disabilities (physical and learning) across five TVET colleges in Gauteng Province, South Africa. Convenient sampling was used in this research using referencing and snowballing techniques from Johannesburg’s five biggest TVET colleges. In addition, five focus groups with various stakeholders involved in the TVET colleges such as the students with various physical and learning disabilities, the staff working at the Student Support Units, lectures, campus managers and government workers working for the DHET. The phenomenological methodology prioritised the voices of the students with physical and learning disabilities studying at the TVET colleges who were given co-researcher status. This resonates with current debates in Disability Studies on the need to co-create knowledge with participants living with disabilities throughout the research process (Chappell & De Beer 2018; Charlton 1998).

Perhaps the highlight of the phenomenological methodology used in this study was the story exercise developed by the researchers together with the students who chose to partake in the study. The ‘story exercise was originally designed by Rooth (1995) as an adaptation of the Participant Rural Appraisal (PRA) techniques first used with disenfranchised groups such as agricultural workers in the United Kingdom as part of a social justice initiative of facilitating the growth of their urgency. The 40 students with disabilities were given the option of participating in a story exercise in which they could narrate their daily activities in various formats, such as voice notes, written notes and picture stories. Twenty of the 40 youths who took part in this study agreed to participate in the story exercise. The 20 young people (from various colleges) who chose to take part in the story exercise were given a blank journal book to fill with pictures, stories, memes and images from magazines, newspapers and social media that best represented their experiences at TVET colleges. The youths were also given a lot of leeway to create stories in whatever format best suited to their abilities, such as pasting pictures, voice notes, drawings, poems, visual arts or simply writing short narratives in handwriting or Braille. The exercises recognise depictions of inclusion, exclusion and disclusion. While the concepts of inclusion and exclusion refer to the clear acts of adding in or leaving out certain groups of people from certain activities, the term disclusion is not so definitive, and it implies when one is neither included nor excluded, a state of precarity when one is expected to be gracious for barely being there on the margins (Moonsamy & Walton 2015). These dynamics occur in everyday interactions and media representations such as newspapers, magazines, social media and television rather than school textbooks and academic journals. The idea is that everyday interactions and incidents in lecture rooms at various TVET colleges (captured in individual stories) painted larger real-life representations of students with disabilities in TVET educational settings. The ‘Data analysis’ section will describe how the data were analysed.

### Data analysis

The researchers sat down with the students who participated to get a subjective interpretation of the picture stories. Then they transcribed and analysed the stories further, weighing down on a decolonial lens of disability. Thematic analysis was then used to break down the analysed data, first into small themes and later merging these into bigger themes (Clarke & Braun 2017). The ‘Ethical considerations’ sections will explore the ethical considerations as well as critically explore the seminal themes that came out of the picture story exercise.

### Ethical considerations

Ethical clearance to conduct this study was obtained from the University of South Africa College of Education Ethics Review Committee (No. 2023/05/10/90525558/19/AM). Signed informed consent was sought from every participant in the interviews, focus groups, story and picture exercises and formal approval letters to conduct research were obtained from the college campuses involved in the study. There was also the use of pseudonyms to protect the confidentiality of participants. The ‘Results’ section will focus on the findings.

## Results

From the 40 students involved in the larger study, 20 students chose to partake in the story exercise and were interviewed to get seminal representations of disability. We then sat down to analyse these self-representations, weighing them against a decolonial lens of disability mentioned earlier in the article. For the sake of this article, we are going to showcase (with the participant’s consent) five of the most memorable stories that came up in the research. We will begin by outlining a small biography for each storyteller and, subsequently, offer a critical analysis of the different stories each participant brought forth.

### Yolanda (albinism)

**FIGURE 1:**
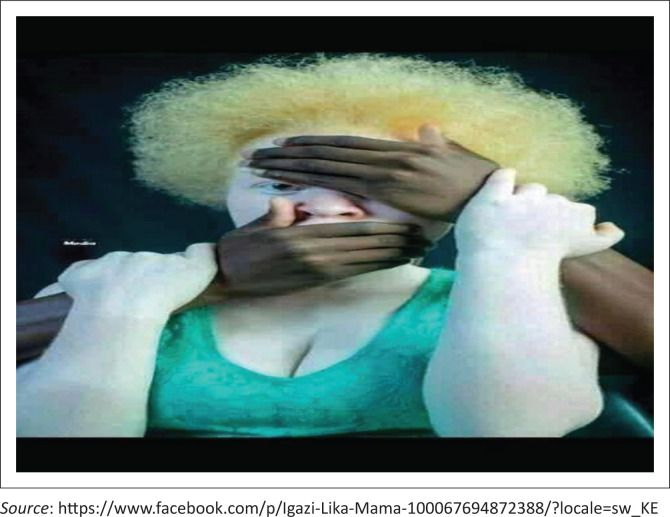
Yolanda’s picture story.

Yolanda is a 20-year-old Xhosa woman with albinism. She is a Business Management student at a TVET college in central Johannesburg. She is from Humphrey in the Eastern Cape and describes herself as ‘authentic, artistic, and open’. She chose poetry (some of which she wrote herself) and images from various social media platforms such as Facebook, Twitter, Instagram and TikTok to represent her stories (with permission from the owners). Here is one of the memorable pictures that she chose to represent her lived experience with albinism in her TVET (Figure 1).

She used this striking picture to tell her story titled ‘Weeping may endure for a night, but joy comes in the morning’. The title of the picture is an extract from Ps 30 vs. 5 in the Bible. One hand is covering one of her eyes, symbolising the concealment of disability by the ableist members of society. Society, when confronted with representations of bodily difference such as albinism, reverts to aversion and concealment of the other fearing what it does not understand. That anomaly should therefore be avoided. One hand is also covering her mouth, symbolising and silencing the other as a form of passive oppression. The hands groping the woman are black, and they stand in contrast to her skin although she is also black. This highlights the plight of the exclusion of people with albinism from their black identity because of skin colour. She narrates a grim story of a girl with albinism who was murdered for ritual purposes, betrayed by people she had trusted: ‘I heard something last year on the news. Someone (living with albinism) was walking with her boyfriend in KwaZulu Natal province, and they told the boyfriend to give them the girl and they would give him money. They found her dead the next day’ [Yolanda’s interview]. Such exclusion is multi-layered as one is already marginalised for being black and, in addition, for not looking black.

### Lebo’s story (learning disability)

**FIGURE 2:**
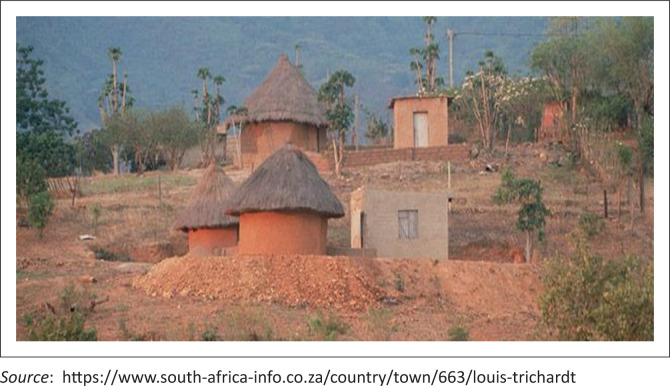
‘Makhado village life’ (Lebo’s picture story).

Lebogang is a 24-year-old student in her second year of studies for a diploma in Educare. She is originally from Makhado, a small town in the South African province of Limpopo. She has dysgraphia, a learning disability that is characterised by impaired writing, which in turn may interfere with learning to spell words in writing and the speed of writing texts. She was diagnosed late in her teens when she was in Grade 10. Her preferred form of storytelling was the use of voice memos and pictures, as she describes herself as having severe challenges with reading and writing. Here is her picture story (Figure 2).

The picture is a representation of Lebo’s home in the mountains of Makhado. She spent most of her childhood and teenage years in this place, and she describes it as peaceful and a breath of fresh air (comparing it to the hustle and buzz of Johannesburg city life). For Lebo, having a learning disability comes more deeply rooted in her African spirituality than in medicalised or socialised discourses. She explains that the circular thatched mud huts have spiritual meaning for her. The circle shapes of the thatched roof are not only symbolic of Africa’s architectural ingenuity. They are spiritual safe houses that offer wisdom and refuge to residents and sometimes even strangers. Lebo loves her childhood home. It is her safe space where she is not bound by any disability labels that threaten to define her. She explains that she often gets misunderstood by her peers and lecturers and her disability is often misinterpreted as sickness, laziness and dullness. She explains that most students shy away from her thinking that her learning difficulties are clinically contagious and can rub onto them as she retorts, ‘Other students feel the need to stay away from me thinking my disability can rub onto them, but my learning disability is not contagious!’ (Lebo’s voice note).

Lebo strongly feels that her learning disability instead has a strong African spiritual root. Her slow learning condition had long been consulted with a local Sangoma (traditional healer) when she was younger. The Sangoma had attributed her inability to learn the Westernised curriculum at her school to a spiritual calling by the VhaDzimu (Ancestors). She needed to heed their calling by performing certain rituals and taking the path of being a Sangoma. An interesting dynamic that played out to further isolate her from her peers is the issue of language and ethnicity. She can only speak her home village language, Venda. This makes her a target of bullying from other students who are mostly local and therefore can speak the local dominant languages, such as isiZulu and Sesotho. Her learning disability, language and dress code pass her easily as eccentric among other students.

Lebo’s story does not end here. She expresses how she has learnt to channel her spiritual energy and her humble upbringing to focus on finishing her college diploma in Educare. She intends to start her preschool after graduating and possibly give back to the family and community that raised her. She later goes on to pass a subject that is considered difficult for students with learning disabilities and by doing so, immediately challenges the narrative that society has constructed around learning disabilities. She begins to command some respect from her peers (some of whom failed the course), and most importantly, she begins to embrace some self-advocacy in her abilities. She was finally on her way to realising her dreams.

### Khensani (dyslexia)

**FIGURE 3:**
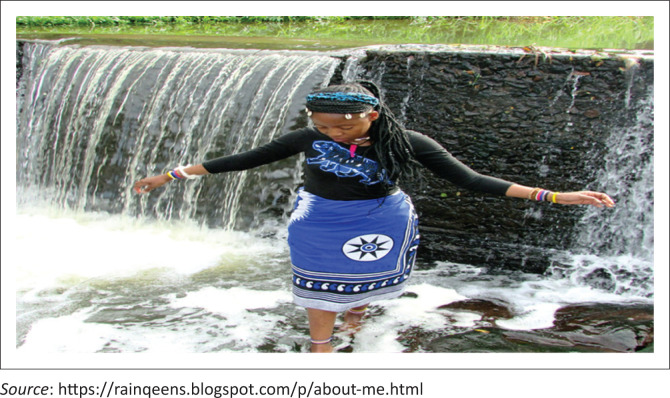
Ubizo (The Calling) Khensani’s picture story.

Khensani is a 29-year-old mother of two girls enrolled for a second year, diploma in Developmental Educare. She lives with her husband in Protea South (Soweto), and she describes him as a loving and supportive father who works full-time while she is studying full-time. She is dyslexic, which creates challenges in reading and writing. Her dyslexia is so severe that she describes experiencing intense anxiety every time she has to explain something in front of the class in the lecture rooms. Some parallels can be drawn from Khensani and Lebo’s experiences with learning disabilities. She received a late diagnosis for her disability (only when she was 25), even though her learning problems started early in her childhood (when she was 2 years old). She cites a lack of awareness around learning disabilities, especially in marginalised communities. She uses a picture story and voice notes to narrate her journey (Figure 3).

The picture in Khansani’s storybook is of a woman dressed in traditional regalia and standing at a waterfall. She explains that it represents her when she was at a Ukuthwasa (a traditional initiation process for someone to become a Sangoma (traditional healer). For Khensani, similar to Lebo, her reading and writing problems have a spiritual basis. Her family believed that she had an ancestral calling (Ubizo) to become a Sangoma (traditional healer). Ideally, she should have gone through an initiation process (Ukuthwasa) a long time ago to connect her to the ancestral world so that she can connect with her healing gift. Unfortunately, Khenzani did not heed this calling for a long time, and this became a basis for her learning challenges. She only accepted the calling when she got registered at the college and found out that she was not coping with her studies.

### Isaac (locomotor disability)

**FIGURE 4:**
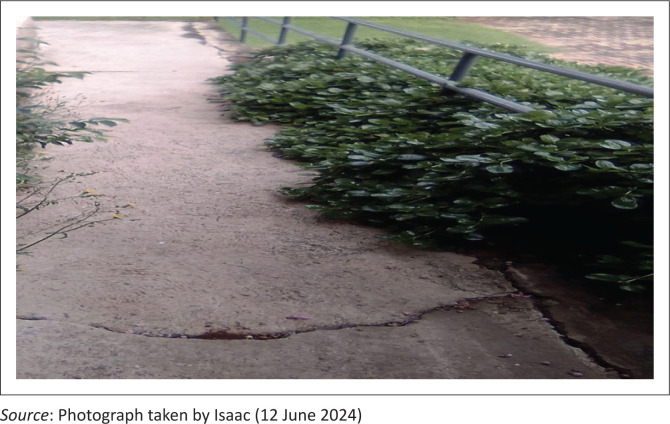
‘Ramp to hell’ (Isaac’s picture story).

Isaac is a 28-year-old student who at the time of the interview was studying for a diploma in Human Resources. He lost both legs after a horrific train accident when he was in Grade 10. He now uses a manual wheelchair. The incident left him depressed as he perceived a loss of independence at first using the wheelchair. However, with time, he got used to it and now describes himself as outgoing and free. He, however, faced many physical built environment accessibility challenges as the TVET college he was attending was not accessible for someone with physical disabilities, especially wheelchair users. For example, the whole college only had one wheelchair ramp, and he often had to rely on other students to carry him when he needed to go up a flight of stairs. He also faced a lot of challenges with accessing public transport as he was staying off-campus, and his picture story narratives and written journal narratives often allude to all these challenges. He also expressed that he initially wanted to study Electrical Engineering at the TVET he was enrolled at, but because the college has not made reasonable accommodations for students with disabilities, he was forced to make amendments and had to settle for a diploma in Human resources. He uses the following picture, which showcases the only wheelchair ramp that is on his campus (Figure 4).

The picture (taken by Isaac on his mobile phone) is titled ‘Ramp to Hell’. Isaac showcases the lack of reasonable accommodation for students with physical disabilities on his college campus. The picture shows a wheelchair ramp that is not only dangerously steep but has also been overrun by bushes. This made it not only difficult but dangerous for a wheelchair user to navigate the college campus. The title of the image is a metaphor for a world filled with ambiguity, as Isaac explains. The rugged ramp, which should have been easing navigation, now adversely acts as an impediment.

### James (Deaf)

**FIGURE 5:**
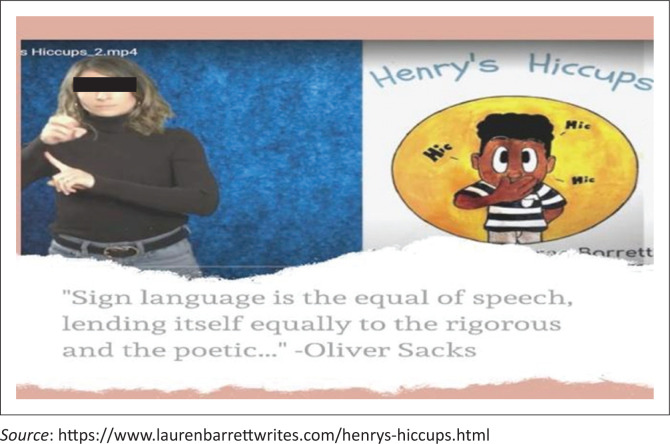
‘Henry’s Hiccups’.

**FIGURE 6:**
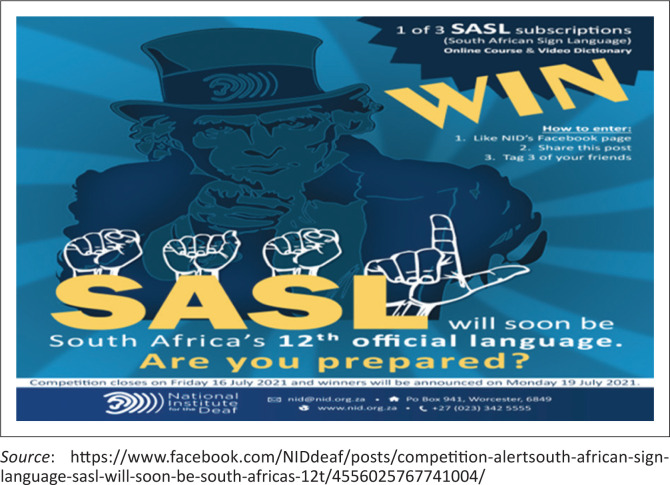
‘Sign language as an official language’.

James is a 23-year-old student studying for a diploma in Administration. He identifies with the deaf (D) culture. He describes himself as a lover of jokes and respects people. He stays in Kleroff, a township in Johannesburg, but he is originally from the suburbs of Florida in Johannesburg. His family moved about 15 years ago because of financial strain when his father lost his job. He describes himself as constantly struggling to fit into his new environment, which is plagued with high rates of crime, alcohol and drug abuse. James chose to use a picture story to narrate his lived experience at the TVET college where he was enrolled. The first pictures he chose are titled ‘Henry’s Hiccups’ (Figure 5) and ‘Sign Languages as an Official Language’ (Figure 6).

The two pictures consolidate South African sign language not only as an official 12th national language but also as a whole cultural identity. The first, titled *Henry’s Hiccups*, is extracted by James from a fictional juvenile book series by Barrett, Pantoja and Etulu (2021). The book series is centred around Henry, who is a happy student until he encounters a hiccup problem in class. He fails to find a remedy, and the hiccups keep getting in his way of communicating with his teacher. He figures out a way of communicating in class using sign language. He began to slowly enjoy the experience of using sign language so much that it became the norm for communicating with his teacher. James’ picture stories bring some very critical perspectives towards Deaf identity among students at TVET colleges in South Africa.

Firstly, it shows that deaf students can assume a culturally hearing identity in sign language through collective stigmatisation by the powerful other (Bat-Chava 2000) (Figure 6). Secondly, it shows that sign language can be taught to young children, and it is possible to normalise it being used as a medium of instruction at any institution of learning in South Africa. Thirdly, as James explains, ‘the pictures also show that everyone can fit into the Deaf culture, irrespective of whether they are deaf or not’. Hence, Henry could learn how to use sign language and feel a close attachment to Deaf culture without being deaf. These findings are like those in Moroe and De Andrade’s (2018) research, which found that hearing children of deaf parents are raised in ‘unique and extraordinary family settings, which makes them culturally deaf (D) yet functionally hearing’ (p. 1). This highlights that a sense of Deaf identity and culture can be constructed at all TVET colleges.

## Discussion

The aforementioned picture stories depict the subjectivity of disability experience among students attending TVET colleges in South Africa. The study showcased how the experiences of students with disabilities at TVET colleges are shaped around the different types of disabilities, that is physical and learning disabilities. For students with physical disabilities, the students’ narratives were centred around a disabling gaze that was often reserved for non-normative bodies (Singer 2012).

For example, with Yolanda, the picture she used is a metaphor and tells her life story of battling against the ableist society that is constantly acting out to mute and conceal her talent and beauty as a person with albinism. She is a part-time model, and she explains how she is constantly wary of the pathologisation gaze whenever she steps on the catwalk or the college campus. To her, society is often shocked that a person with albinism can also be beautiful, intelligent and a successful model. She is often bombarded with praise for her strong confidence and how her skin is blemish-free ‘unlike other people with albinism’ (Yolanda, picture story narration), and such incidences could be said to be reinforcing the concept of inspiration porn. The term *inspiration porn* was made popular by Australian disability activist and comedian Stella Young (2014). It refers to controversial representations of disability as a disadvantage and tragedy that can be overcome, often for the gratification of an able-bodied audience (Grue 2016). Seen in this light, in the case of Yolanda, *inspiration porn* could be said to be manifesting itself in the form of objectifying and sexualising her, and she is often left uncomfortable.

Furthermore, closely linked to the clinical gaze is the act of concealment and looking away. Yolanda narrates how other sponsors of her brand sometimes look away when she is modelling because her colourlessness represents bodily differences outside of the norm in the modelling industry. However, as the title of the picture suggests, this is just the beginning of her narrative. In the picture, the girl is also clutching at the intruders’ arms in defiance. She is not passive or hopeless and she is fighting back. Similarly, Yolanda describes herself as constantly fighting to control her narrative set against the ableist college environment and world.

Most students with albinism in the study demonstrated a high level of agency, creativity, autonomy and motivation to improve their lives, only becoming debilitated through discrimination by the ableist society (Kiishweko 2017). For Yolanda, being the only student with albinism on her college campus came with its perks of being celebrated as exotic and interesting on campus. On the first day on campus, everyone wanted to be her friend, and she felt genuinely welcomed by the whole college. However, this turned out to be a double-edged form of appreciation as she soon found out about the killings of people with albinism that were occurring in her community.

In this instance, the exclusion is symbolised through the cultural representation of witchcraft and superstition surrounding people living with albinism in sub-Saharan Africa (Mulemi & Ndolo:^2014^). For Yolanda, the image of TVETs as safe havens for people with albinism is distorted by the infiltration of the bio-cultural exclusion on her campus. She is haunted by the images of people like her on social media platforms who disappear, targeted by ritual killings or the desecrated images of graves exhumed to extract umuthi (magical portions).

The difference and stigma that come for students with physical disabilities such as albinism in South Africa, therefore, transcend the body and the TVET campuses to merge into a more vicious form of genocide, a threat to a whole civilisation and cultural identity of people who have been labelled the other, simply by the colour of their skin.

### Ignorance and suspicion

The experiences of studying at a TVET college for students with learning disabilities are on the other spectrum surrounded by an air of ignorance and suspicion from an ableist society. Such suspicion evolves around the medical discourses of contamination as in the case of Lebo and Khensani. There is also a cultural and spiritual element attached to learning disabilities, whereby spiritual unfulfillment is associated with difficulties in learning. However, such challenges in learning and life in general because of unfulfilled ancestral callings are not unusual in a Southern African context. It is often a well-known belief in African culture that it is taboo for a person to deviate from their ancestral calling. It is also a well-known belief that a person can also have unexplained mental illness, and nothing will work out for them until a time they choose to reconcile with their ancestors’ wishes. For example, Bakow and Low (2018) posited that ancestral calling has been compared to Western mental health constructs, such as psychosis because of the accompanying symptoms. Khensani, later, gave updates on her experiences with a learning disability since she had the Ukuthwasa initiation. She said she gained a lot of self-confidence in dealing with her learning disability. Part of the reason that she faced intense anxieties around other people at the college before she got initiated was that she lacked a sense of cultural identity and a spiritual sense of purpose in life. Now that she is a Sangoma, she has learnt to channel her spirituality to connect and control her learning anxieties.

The idea of using African traditional thought and culture in conceptualising and dealing with disabilities is not a new one. It falls in line with African traditional theoretical frameworks such as the Ubuntu philosophy. Scholars such as Menkiti (2017) and Gumbo, Gasa and Knaus (2022) continually advocate for an African cultural sense that is a whole way of life and that can be used to construct and reconstruct how African people conceptualise and adjust to university life. Spirituality is the medium of that culture that helps Khensani and Lebo to deal with their learning disabilities.

### Compulsory able-bodiedness

The study also exposed how the notion of integrated learning in public TVET colleges still resides in an ableist, misogynistic and hetero-normative notion of society that still largely perceives an engineer or a technology expert as someone able-bodied and male. This is highlighted by Isaac’s story of having to change his career path because of a lack of reasonable accommodation in some of the curricula at his college. Article 2 of the UN Convention on the Rights of Persons with Disabilities (2006) defines reasonable accommodation as:

Necessary and appropriate modification and adjustments not imposing a disproportionate or undue burden, where needed in a particular case, to ensure to persons with disabilities the enjoyment or exercise on an equal basis with others of all human rights and fundamental freedoms. (p. 2)

Concerning the right to education, reasonable accommodation means ensuring the specific support needs of students with disabilities are provided so that they can equitably participate in learning alongside their peers, which was unfortunately not the case with Isaac. He explains that:

‘They (Student Support officials) advised me to make registration amendments and chose a different diploma because Electrical Engineering did not offer reasonable accommodation for someone in a wheelchair. They said I am the first student at the college who was using a wheelchair and wanted to do Engineering so they were making amendments to the curriculum so that it can accommodate someone like me!.’ (Isaac’s interview)

Compulsory able-bodiedness (McRuer 2006) involves the essentialist assumption that an able body is the one that is considered ‘normal’ and what Garland-Thomson (1997) refers to as the ‘normative body’. Inahara (2009) unpacks further and states that ‘It implies that all human beings are represented by only one body, which is able’ (p. 47). We argue that compulsory able-bodiedness is a problematic social construct in TVET settings because it erroneously expects every student who enrols in public TVET colleges to be able-bodied and heterosexual if they are to be accepted by the curriculum and society as ‘normal’ and be able to handle the training and apprenticeship aspects.

### Disability through the lens of intersectionality

Based on the findings, it could be argued that a critical lens is needed that examines the intersections of race, class, gender, sex and masculinity in disability. Some critical disability studies scholars (see Goodley 2011; Sherry 2016) have argued that disability should be understood in the context of other multiple identities and should also be placed at the centre of them. In this study, the theme of intersectionality played out when examining the demographics of the students who participated. The 40 students with disabilities who participated in the overall study were predominantly of African, mixed race and Indian heritage. This racial disparity is not by coincidence. To understand the current situation of adolescents and young people with disabilities in public TVET colleges in South Africa, one needs to gain insight into a comprehensive historical overview of TVET education in South Africa as a legacy of colonialism, apartheid and capitalism (Masuku & Hlela 2023). There was a need for cheap labour under the apartheid system and vocational education was considered the first education to be received by Africans from the colonists, especially from the British model (Masuku & Hlela 2023). This legacy has persisted long after Apartheid as TVET education is still viewed as black as highlighted by the demographics of the students in this study.

This means that disability experience of the students at TVET colleges cannot be understood outside other identities because there is always an intersecting orientation and belonging to a particular class or race by persons with disabilities. Thus, disability might not be understood in isolation from those other identities. Sherry (2016) further argued that disability should be understood as ever-changing and fluid, shared by people with and without disabilities. What Sherry (2016) meant can be understood from other scholars’ different perspectives of understanding disability. For example, Puar (2015) stated that ‘You are able-bodied until you are disabled’ (p. 149). That implies the fluidity of disability; that there is a thin line between those with and without disabilities because the former could also be disabled in one defining moment. Thus, disability should be understood as a continuum rather than a binary of able or disabled. People with disabilities are not to be viewed as a group on their own with special needs but as ‘diver-sea-ty’ (Steyn 2011), among other diversities in people with and without disabilities. As Goodley (2011) points out, ‘disability should be seen as a springboard: space from which to think through a host of political and theoretical issues that apply to all identities’(p. 185). In the context of this research, understanding intersectional disability identity will help to understand students with disabilities learning at TVET colleges as an integral part of the diverse student body in higher learning. The ‘Contributions and implications’ section offers the contributions of the study towards an evidence-based framework for promoting disability equity for indigenous populations.

## Contributions and implications

Firstly, the article has added new knowledge in the field of disability inclusion in higher education especially in relation to previous/existing scholarly literature on the South African TVET sector. There are few, if any TVET studies that have employed a unique methodology such as the pictures story used by the authors that emphasises the co-creation of knowledge with disabled students’ participants using images.

Secondly, this research also makes recommendations for evidence-informed action plans aimed at promoting disability inclusion in TVET colleges from the Global South to make use of participatory research methodologies with indigenous peoples. Participatory research with indigenous peoples ‘prizes partnership between individuals (and/or the groups they represent) who have a stake in the research, including (but not limited to) Indigenous peoples and researchers’ (Dadich, Moore & Eapen 2019). This partnership involves equal opportunities for engagement between different individuals. Participatory research with indigenous peoples is recommended for a better understanding of public health. Collaborating with indigenous communities requires clear evidence of active participation and equal ownership of research. In summary, promoting equity, cultural safety and contextually tailored care are essential for improving health services for indigenous populations. While specific resources related to disability inclusion in TVET colleges may be scarce, these principles can guide future research and policy efforts.

Thirdly, evidence-informed action plans aimed at promoting disability inclusion in TVET colleges from the Global South should be guided and informed by indigenous knowledge systems (IKS). The concept of IKS emphasises the importance of traditional knowledge held by indigenous communities. While not directly related to disability inclusion, it highlights the value of incorporating indigenous perspectives in research and policy.

### Limitations

The applicability of IKS to the inclusion debates comes with its share of challenges and hurdles. There have been sceptics who have criticised the ‘return’ narrative of indigenous epistemologies such as Ubuntu as frivolous and no longer applying to South Africa and most countries from the Global South (Matolino & Kwindingwi 2013). These scholars equally doubt Ubuntu’s validity and global applicability in producing Western equivalents. Simply put, these critics doubt if indigenous knowledge can be used to explain contemporary phenomena as it is outdated, tumulous and dying (Matolino & Kwindingwi 2013). Major arguments put forth by these scholars are that indigenous knowledge faces a formidable foe in the form of capitalism, which they feel is deeply rooted even in the so-called communist countries that it would be a next-to-impossible fit to try to outlaw it. In addition, IKS such as Ubuntu are also deeply inclined towards the conceptual, leaving little room for applicability – and sometimes even breeding tension and conflict. A case in point is the high incidence of hate crimes, organised crimes and xenophobic attacks in South Africa, which have been used conveniently as a measure of the limitations and failures of IKS (Koenane & Olatunji 2017). Similarly, practices such as the killings of people living with albinism in sub-Saharan Africa have been used to discredit IKS when it comes to how communities deal with persons with disabilities.

However, proponents of IKS such as Koenane and Olatunji (2017) point out that Ubuntu philosophy is not dying but is birthing into a theory with ‘greater prominence than other rival theories’ (p. 265). This is because Ubuntu is an authentically African belief system that has managed to morph well with different sub-cultures across the continent. The authors give the example of Nyerere’s Ujamaa in Tanzania, Ubuntu in South Africa, Unhu/Ubuntu in Zimbabwe, Umuchinshi in Zambia, Botho in Botswana and the Gada philosophy among the Oromo people in Ethiopia as all successful variants of IKS. To simply discredit African indigenous knowledge based on a few incidents is to automatically nullify ‘all strong theories and systems of today that have evolved through debates, suggestions, criticisms and contributions, not by ceasing to discuss and challenge them’ (Koenane & Olatunji 2017:266).

## Conclusion

These are narratives of the students and many more that could not be added because of the limitations of time on this study. They should make society, lecturers, management, researchers and academics working in the TVET space reimagine and rethink their day-to-day interactions with various disabilities. Most importantly, these narratives forewarn and challenge disability inclusion scholars in education to be wary of dominant epistemologies of disability representations – that often act out to reinforce troublesome stereotypes that have been associated with people living and learning with disabilities in TVET colleges. From a decolonial perspective, the experiences of having a disability while studying at TVET colleges are clouded by issues of poor access to the architectural landscape and curriculum of colleges by students with disabilities the majority are also black. This brings out the issue of the intersectionality of disability – whereby the experiences of marginalised identities who are living with a disability are not necessarily the same with other ethnic groups.
